# Role of Barrier Integrity and Dysfunctions in Maintaining the Healthy Gut and Their Health Outcomes

**DOI:** 10.3389/fphys.2021.715611

**Published:** 2021-09-24

**Authors:** Shruti Panwar, Sapna Sharma, Prabhanshu Tripathi

**Affiliations:** ^1^Infection and Immunology, Translational Health Science and Technology Institute, National Capital Region (NCR) Biotech Science Cluster, Faridabad, India; ^2^Gene Regulation Laboratory, School of Biotechnology, Jawaharlal Nehru University, New Delhi, India; ^3^Food Drug and Chemical Toxicology Division, Council of Scientific and Industrial Research (CSIR)-Indian Institute of Toxicology Research, Lucknow, India

**Keywords:** tight junctions proteins, metabolic disorders, phytochemicals, membrane integrity, intestinal epithelial barrier

## Abstract

Mucosal surface layers are the critical borders throughout epithelial membranes. These epithelial cells segregate luminal material from external environments. However, mucosal linings are also accountable for absorbing nutrients and requiring specific barrier permeability. These functional acts positioned the mucosal epithelium at the epicenter of communications concerning the mucosal immune coordination and foreign materials, such as dietary antigens and microbial metabolites. Current innovations have revealed that external stimuli can trigger several mechanisms regulated by intestinal mucosal barrier system. Crucial constituents of this epithelial boundary are physical intercellular structures known as tight junctions (TJs). TJs are composed of different types transmembrane proteins linked with cytoplasmic adaptors which helps in attachment to the adjacent cells. Disruption of this barrier has direct influence on healthy or diseased condition, as barrier dysfunctions have been interrelated with the initiation of inflammation, and pathogenic effects following metabolic complications. In this review we focus and overview the TJs structure, function and the diseases which are able to influence TJs during onset of disease. We also highlighted and discuss the role of phytochemicals evidenced to enhance the membrane permeability and integrity through restoring TJs levels.

## Introduction

The gastrointestinal inner membranous arrangements sketch out the human body's principal boundary with the outside world. The epithelium permits the nutrient absorption, supporting a physical barrier to the dispersal of proinflammatory components, like microorganisms, foreign antigens and toxins from the luminal milieu to the adjoining mucosal tissues (Groschwitz and Hogan, 2009). The epithelial membrane constructs selective permeability by either transcellular or paracellular methods. In transcellular mode of permeability, micronutrients such as carbohydrates (like glucose, hexose, and fruits etc.), amino acids, small size proteins, fatty acids, minerals, and vitamins are absorbed and transported. Specific channels or transporters situated on upwards (apical) and downwards (basolateral) sides of cell membranes plays a crucial role due to impermeability of membrane (Kapus and Szászi, 2006). In contrast, the paracellular mode is related with transport through the intercellular gaps among the bordering epithelial cells. This is controlled by combination of adherence junctions (AJs) and tight junctions (TJs) forming the junctional complex (Kiela and Ghishan, 2009; Suzuki, 2013). TJs are compulsory for maintaining a barrier among different sections of the body. The primary function of these TJs are to act as interstitial space for entry limiting the charge and size-based diffusion. Selective paracellular diffusion is a vital mechanism for the continuous homoeostasis among tissues. TJs have not studied on functional levels and considered as most mysterious of all adhesion complexes and have limited molecular and functional investigations due to their complex structures. In recent years proteins associated with tight junctions have been identified with different functions. These studies have confronted the conventional model where the tight junctions were considered to be merely a barrier controlling the diffusion of macromolecules. In addition to multiple functions, tight junctions are also associated with several human diseases especially due to their mutations at genetic level. Furthermore, tight junctions encounter countless viruses, bacteria, and foreign antigens, they affect functional and signaling behavior of TJ components for crossing the barriers. TJs are considered as preserved feature, with their biological functions across the vertebrate during evolution. The main objective of this review is to comply the current inventions about the biological functions of tight junctions and their role in various metabolic disorders. We discussed the understanding of how tight junctions regulates as signaling strategy that reflects the cellular response toward disease progression.

## Cellular Constituents of the Mucosal Barrier

**Figure 1:**
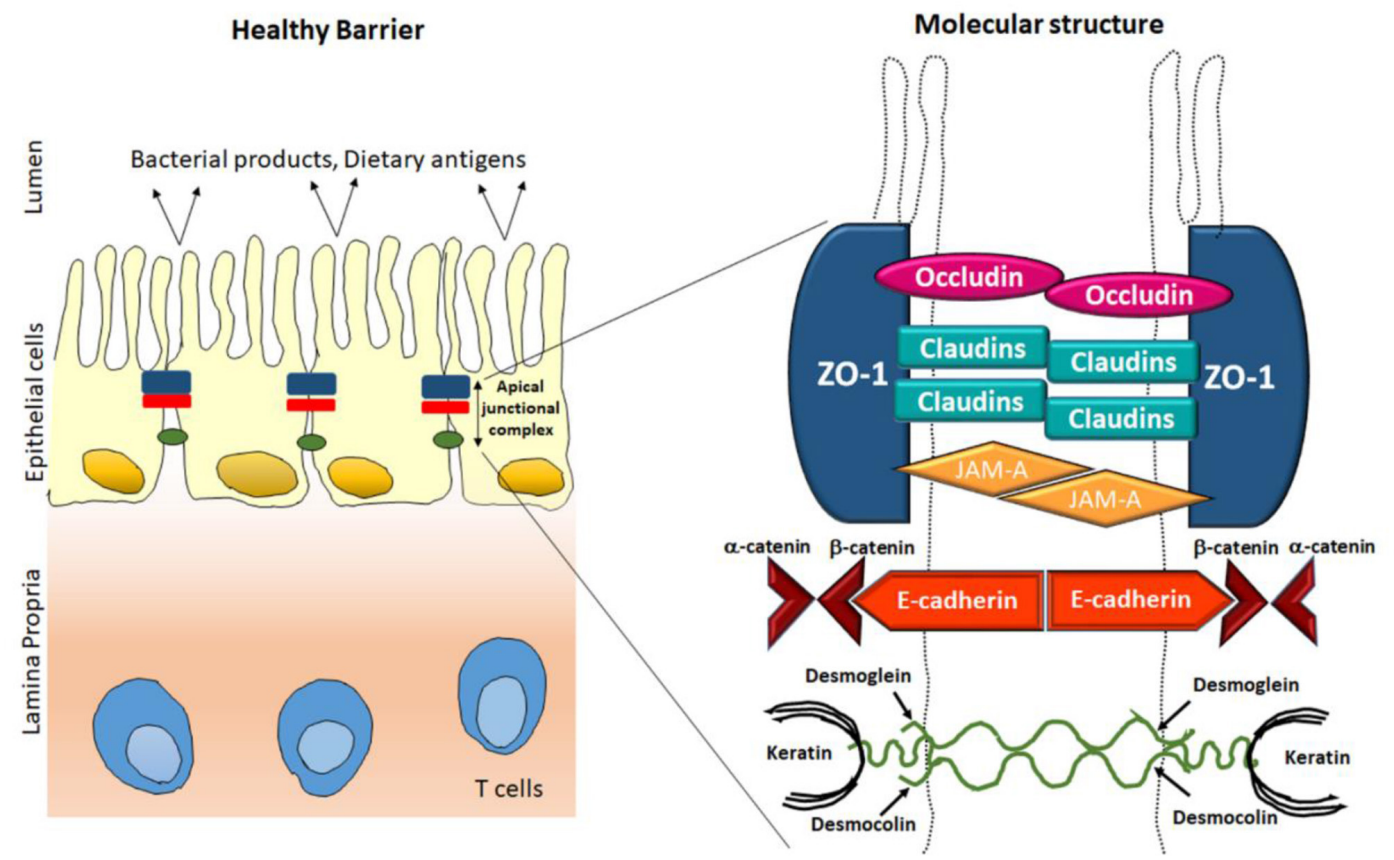
Classical overview of epithelial tight junction's structure in healthy conditions.

The main concern for barrier function at mucosal surfaces inhabits with the plasma membrane of epithelial cells, which is resistance to many hydrophilic solutes due to lack of specific transporters. There are many direct exposures which induce damage to epithelial cell for example, mucosal antigens or cytotoxic agents, such as drugs used for chemotherapy during cancer treatment result in a significant loss of barrier function. Along with the existence of a firm epithelial monolayer, the paracellular pathway between cells must be preserved and functions well. This function is regulated by the apical junctional complex, an assembly of the tight junction and adjacent adherens junctions (AJs) (Figure 1). The adherens junctions and the desmosomes give robust connective integrity among the epithelial cells. They also encourage intercellular interactions, but cannot regulate paracellular permeability (Brooke et al., 2012). The tight junctions are present on the apical side of the epithelial cell membrane and regulates the solute permeability paracellularly (Lee, 2015). In this respect, the tight junctions offer dual role, barrier as well as a pore, which are generated for the permeation of tiny molecules. Tight junctions are protein multiplexes composed of different proteins such as transmembrane proteins, claudins, occludins and lots of proteins in the cytosol. There are several extracellular stimuli that alter TJ barrier functions and paracellular permeability and are linked with disease vs. healthy conditions (Harhaj and Antonetti, 2004). Compromisation in tight junction barrier with augmented paracellular permeability result in translocation of proinflammatory factors in the lumen, encouraging mucosal immune activation, which subsequently lead to tissue injury and inflammation. Scientific studies and clinical observations suggest that the intestinal barriers including TJs and AJs have crucial role in in the pathogenesis and progression of intestinal as well as systemic diseases (Buckley and Turner, 2018). Under pathophysiological circumstances, pro-inflammatory cytokines, foreign antigens, and harmful microorganism contribute to barrier diminishing (Chelakkot et al., 2018). On the other hand, many food factors and nutrients also added in intestinal TJ regulation, and few possibly developed as prophylactic and therapeutic tools for compromised barrier-related disorders (Bischoff et al., 2014). TJ barrier integrity and permeability in *in vitro* models are measured by determining transepithelial electrical resistance (TER) and the strength of paracellular channels for small molecules like mannitol, dextran, and inulin was studied using permeability efflux. This review covers the structural component of apical junctional complex and summarizes the role of tight junction in regulating the barrier permeability and its relation with health consequences. This review also discusses about the selective participation of phytochemicals among intestinal TJs in well-being and disease progression.

## Structural Overview

TJs are manifold protein multiplexes positioned on the intestinal epithelial cell membrane at the apical side (Figure 1). They control paracellular permeability by regulating the passage of water, ions, and other solutes. TJs act as a boundary for macronutrient like lipids and proteins between basolateral and apical side of plasma membrane by blocking their free diffusion, this and preserve the cell polarity (Anderson and Van Itallie, 1995). There are four integral proteins, popularly known as occludin (Cummins, 2012), claudins (Krause et al., 2008), junctional adhesion molecule (JAM) (Ebnet et al., 2004), and tricellulin (Koval, 2017), among them claudin family comprising almost 24 more members. The neighboring cells form the barrier by hemophilic and heterophilic communications with the help of extracellular domains of transmembrane proteins (Tsukita et al., 2001; Ding et al., 2013). The intracellular domains of the transmembrane proteins cooperate with complex proteins in the cytosol, like zonula occludens (ZO) which further binds the transmembrane proteins to the internal junctional actomyosin ring. This interaction between TJ proteins along with actin cytoskeleton is necessary for the TJ structural upholding and functionality of TJs. Myosin light chain (MLC) activity (phosphorylation) regulates the circumferential narrowing and firmness of the perijunctional actomyosin ring (Cunningham and Turner, 2012). Kinases like Rho-associated kinase (ROCK) and myosin light chain kinase induces the MLC phosphorylation which leads to contraction of the actomyosin ring, subsequently opening the paracellular pathways. This segment recapitulates the structures and roles of the integral TJ proteins (Du et al., 2016; Jin and Blikslager, 2020).

### Peripheral Membrane Adapter Proteins

#### Zona Occludin

The ZO proteins were the primary to be studied as TJ-specific proteins, till now three isoforms exists, popularly known as ZO-1, ZO-2, and ZO-3 (Itoh and Bissell, 2003). Sequence resemblance analysis, determined that ZO proteins belongs to the membrane-related guanylate kinase homolog family (González-Mariscal et al., 2000). They are multiple domain proteins comprising three post-synaptic Z domains, a Src homology-3 domain and a area of homology to guanylate kinase adjacent to N-terminus (Heinemann and Schuetz, 2019). These multiple domain assemblies deliver an inner cellular framework in the TJs and are crucial for regulation and structural preservation of TJ structure. As illustrated in Figure 1, the ZO proteins associate with other proteins, like actin and its related proteins to establish a solid framework for maintenance of membrane integrity. Previous reports stated that there are mutual interactions among ZO-1, ZO-2, ZO-3, but interestingly ZO-2 and ZO-3 do not interact with individually (Paris et al., 2008). ZO-1 coordinates with ZO-2 and ZO-3 within the cell, in the presence of the another PDZ domain, PDZ behave as a primary domain in anchoring receptor proteins within the membrane to internal cellular structural components (Umeda et al., 2006). There are reports that in MDCK cells, tight junction comprises of ZO-1 resides together with both ZO-2 and ZO-3. Even ZO-1 and ZO-2 combines with each other, the presence of ZO-1 interacts to a ZO-3 affinity column does not show the holding of ZO-2, thus demonstrating that ZO-1 does not engage ZO-2 with ZO-3 (Nomme et al., 2015). Among the ZO proteins, ZO-1 is the most studied protein in last two decades. ZO-1 confines to the promising cellular interaction both in *in-vitro* models as well as in *in-vivo* models (Tornavaca et al., 2015). Hence, it has been recommended that ZO proteins may facilitate the primary assembly of TJ proteins into cell-cell contacts. Till now, exhaustive attempts were performed to explore the structural role of ZO proteins but the functional role is still not clear. Although several researches have made attempts their attempts to confirm its function in ZO-1 deficient cells (Guillemot et al., 2008). They have created epithelial cells deficient of both ZO-1 genes and these ZO-1-deficient clones are still able to express healthy TJ structures and exhibited regular permeability; however, noticeable pause in the formation of TJ assembly comprising occludin and claudins was detected, demonstrating that ZO proteins have an imperative responsibility in initiation and regulation of this complex assembly (Umeda et al., 2004).

### Integral Membrane Proteins

#### Occludin

Occludin, the foremost recognized module of tight junction threads was discovered by Furuse et al. (1993) and named after the Latin word “occlude.” Structural analysis of membranous protein determined that it consists of 4 hydrophobic membrane bridging domains, two extracellular 44 amino acid loop and two intracellular domains. The initial extracellular loops of amino acid arrangements are primarily rich in two residues namely, glycine and tyrosine. Sequencing provides more decisive evidence about the occluding structure. It is assumed that almost, two extracellular loops have 60-kDa tetraspan membranous protein whereas, a small intracellular turn, and cytoplasmic domains having N- and C-terminal (Furuse et al., 1993). The entire NH2 terminal which is half of the occludin protein, is categorized into five different domains (domains A-E). The half portion of –COOH terminal also known as domain E in cytoplasm is comprised of ~250 amino acids, constrain and is highly enriched with charged residues (Furuse et al., 1994). Among these extracellular loops about 60% are extremely rich in amino acids such as glycine and tyrosine and minimally are charged amino acids. In a same way the C-terminal regions are rich in amino acids such as threonine, serine and tyrosine. These amino acids are commonly phosphorylated by several type of protein kinases which are present in both the sides (apical as well as basolateral) of the cells (Sallee and Burridge, 2009). Additionally it was also documented that, the C-terminal sequence was bounded directly to ZO-1 protein (Tornavaca et al., 2015), which indirectly links with the actin cytoskeleton (Odenwald et al., 2017). Though, occludin isoforms have been seen (Mok et al., 2013), and there is no clear variance in their tissue distribution and functions has been demonstrated yet. Its expression co-relates to a lot many pathological diseases including diabetes which is associated with gut channels (de Kort et al., 2011). Diabetic rats experienced lowered expression of occludin levels but no change was found in ZO-1 levels (Chehade et al., 2002). It has also been associated with blood-retinal high VEGF, since the increase in permeability was observed due to the lowered expression of occludins (Antonetti et al., 1998).

#### Claudin

Claudins are family of proteins along with occludins that forms the most significant structural part of TJ complex zona occludins. Claudins word originates from “Claudere” which indicates near. They maintain the cell-cell connection among plasma membranes of nearby cells and to regulate the para cellular flow of ions (Krause et al., 2008). Claudins are ~25 kD transmembrane protein, reach over the membrane four stages and lies within both N & C terminal within the cytoplasm (Rüffer and Gerke, 2004). The claudin protein family in mammals constitutes 27 proteins that are essential for maintaining paracellular barrier in membrane of epithelial cells. All the members of claudin family consists of similar common structural skeleton, consisting cis- and trans- interactions among them but have diverse extracellular regions that are responsible for different functions in creating paracellular paths and channels. Human claudins are composed of approximately more than 300 amino acids which have a molecular weight varing from 21 to 34 KDa (Günzel and Yu, 2013). The basic structure of claudins have an intracellular short –NH2 terminus (except for claudin−5,-16, and−25), a long intracellular –COOH terminal, 2 extracellular loops (ECL1 and ECL2) and one tiny intracellular loop. The predicted ECL1 resides between the first uppermost and second layer of transmembrane helices and is a sustained loop of ~42–56 residues. It forms the lining of paracellular pore for transport of small ions due to its charge selectivity property. ECL1 also contains a conserved signature sequence of GLW which may play an important role in claudin structure stabilization and hence stabilizing trans interactions (Krause et al., 2009). ECL2 has a conserved amino acid sequence and is important for trans interactions. Claudins are organized in membrane as 4 transmembrane proteins that contain two extracellular parts (ECS1 and ECS2 in mouse claudin-15) arranged in beta sheets of five elements (β1–β5) adjacent to the upper surface of membrane. The extracellular beta sheet becomes stable with consensus motif, delivers an interface for intracellular adhesion. Claudins presents in adjacent cells have variable regions V1 and V2 and are important for communication. The cis interaction is hydrophobic in nature and provides the protrusion of extracellular helix and pocket of the extracellular region of transmembrane 3 (TM3) of neighboring claudin molecule is known to form the claudin cis-polymerization resulting in tight cell-cell adhesion. Claudins are pore proteins depending on variability in epithelial ionic permeability which is measured in terms of transepithelial electrical resistance (TEER). They play a crucial role in charge- and size- selective conductance of molecules in a paracellular manner. It is an important constituent of TJs and is known to have altered expression in different pathological conditions. One such example is diabetes which is accompanied by oxidative stress and hyperglycemia and, which is a main cause of damage in kidney. Diabetic renal damage is a popular complication and includes different functional variations such as natriuresis (Komers and Cooper, 1996), hyperfiltration (Mogensen, 1971) andproteinuria (Najafian and Mauer, 2009). Proteinuria is used as an early marker for detection of diabetic nephropathy (Najafian and Mauer, 2009). In one of the studies, it has been demonstrated that claudin-5, a constituent of glomerular barrier was shown to have decreased expression which might be the reason of early proteinuria in diabetic condition. On the other hand nitration of claudin-2 was shown to be a novel mechanism for increased natriuresis in diabetic rats (Molina-Jijón et al., 2014).

#### JAMA

JAM is a 40 KDa molecule which was first identified using monoclonal antibodies raised against endothelial cells (Martìn-Padura et al., 1998), albeit it is also present in epithelial cells. JAM- type A, type-B, and type–C belong to an immunoglobulins family and are type 1 transmembrane proteins containing extracellular immunoglobulin (Ig)- like (distal domain-D1 and proximal domain-D2), single transmembrane spanning domain, and a cytoplasmic domain which ranges from 40 to 48 amino acids. They have two common structural motifs necessary for biological activity. The first motif is present in the Ig domain D1 and has a signature tripeptide containing two differently charged residues of amino acid detached with hydrophobic amino acids. For JAM type-A, this motifs facilitate homodimerization in cis, through formation of salt bridges by differently charged amino acids in the among two monomers (Ebnet, 2017). The structure of JAM type-A crystal also expects the presence of additional residues in D1 domain responsible for cis-dimerization. All the three JAM proteins consist of –COOH terminus at canonical PDZ domain which forms network with several other scaffolding proteins such as ZO type-1, ZO type-2, Afadin, Par3, and MUPP1 to assemble signaling complex. Epithelia forms a barrier to avert flow of water, tiny molecules and microbes across epithelia layers. The important structure at epithelial tight junctions accountable for maintaining barrier function is Tight Junctions (TJs). JAM-A is rich in Epithelial cells, but not specifically restricted at TJ. The magnitude of JAM-A in maintaining epithelial barrier and paracellular permeability for tracers is well-understood. These specific functions are mediated by localization of serine 285 phosphorylated pool of JAM type-A at junctional point after setting up mature cell-cell contacts through interaction of PAR-αPKC complex and spot like adherens junctions (pAJs).

#### E-Cadherin

Cadherins are the part of calcium dependent cell surface adhesion protein family. Classical cadherins such as N-, P-, and E- cadherin were first identified in vertebrates (Takeichi, 1988), known to be localized in adherence junction to form an adherence belt and build an interaction with actin containing cytoskeleton. The human E-cadherin is a glycoprotein of molecular weight of 120 KDa which is transcribed by CDH1 gene, located on the chromosome 16q22. It is constituted by a transmembrane segment, one large extracellular domain, and a short cytoplasmic domain. There are five tandem repeats of 100 amino acids comprising an extracellular domain and among them N-terminal repeat has the largest part with adhesive functional activity.

Such portions of the molecule possess sites for calcium binding. The cytoplasmic tail interrelates with cytoskeleton framework through actin proteins such as alpha-, beta-, and gamma- catenins and is functionally important as it supports cadherin clustering and provides adhesive support (Shapiro and Weis, 2009). Cell-cell adhesion is important for providing a physical anchorage system to form a stable and highly organized tissue structure. Also, these contacts allow for effective intercellular communication and establish transmission though intracellular pathways with the help of macro and micro molecules present in plasma membrane. E-cadherin is primarily involved in formation of adherens junction and thus plays a crucial role in establishing the homeostasis of the healthy adult epithelial tissue for structure and integrity balance (van Roy and Berx, 2008). It has been reported that soluble E-cadherin, found in patient's urine with diabetic nephropathy and can act as a potential biomarkers for its early detection. The marker was identified using a proteomic based analysis of urinary E-cadherin of patients having type 2 diabetes with normo-albuminuria microalbuminuria, macroalbuminuria and is upregulated 1.3-, 5.2-, and 8.5-folds, respectively as compared to control group (Jiang et al., 2009). In another study of maturity-onset diabetes of the young population, it has been observed that pancreatic islets show abnormal framework and lowered expression of E-cadherin. E-cadherin mediates cell adhesion which is responsible for eliminated glucose which further leads to upregulation of intracellular calcium and insulin secretion, indicating that impaired insulin secretion is due to loss of E-cadherin (Yamagata et al., 2002).

#### Cingulin

Cingulin (140 kDa) is an internal protein of the TJs and recognized as a cytoskeleton-related actin protein. Investigators claimed that these proteins form a homodimer with huge N-terminal head and tiny globular C-terminal tail with coiled rod domain (Citi et al., 1991). Previous reports demonstrated that cingulin is located at the internal side of TJs and interact with many other TJ proteins, representing its part in TJ complex through GATA-4 signaling (Guillemot et al., 2013). Though its functional position is not known completely, studies has shown that the head domain connects with ZO proteins (Hou, 2019). In addition, *in vitro* binding assay discovered that there is a direct binding to occludin and JAM-A (Ebnet et al., 2004). Reports have also demonstrated that disruption of the gene for cingulin doesn't prevent the formation of tight junction rather alter the expression and levels of protein (Guillemot et al., 2004).

## Clinical Significance of Intestinal TJs Proteins in Metabolic Diseases

The intestinal epithelial TJs create a corporeal fence against luminal antigens and molecules such as pathogenic bacteria, toxins, and food allergens. Higher diffusion of such molecules over the distorted barrier can result in pronounced initiation of inflammation and immune system. Consequently, barrier deficiency is carefully linked with the pathology of various gastrointestinal and systemic inflammatory disorders. Although various elements are engaged in disease initiations, barrier dysfunction can be one of the consequences leading in the direction of disease progression. Evidence from past two decades on basic scientific and clinical research suggest that barrier dysfunctions can play important role in the initiation and disease progression. This final portion of this review focusses on experimental studies about the association of intestinal TJ barrier dysfunctions and disease development and progression.

### Possible Role of TJs in Different Disease Conditions

#### IBD

Includes many gastrointestinal disorders such as ulcerative colitis and crohn's disease. IBD progresses with intensive inflammation due to affected barrier integrity which may lead to the mislaying of solutes and essential fluids beyond the barrier. This results in high amounts of antigen translocation which is the major reason of immune cells flux and inflammation causing the barrier to disrupt further (Holmberg et al., 2018). Ulcerative colitis and crohn's disease are linked with high rate of loss of epithelial cell through apoptosis (Di Sabatino et al., 2003) which relates the immune response to cell death. Constituents of epithelial barrier affected in such diseases are TJs, mucusal layer, and antimicrobial peptides.

#### Crohn's Disease (CD)

Increased expression of Claudin 2 and reduced redistribution of Occludin, Claudins like−3,−5,−8. Cytokine profile consists of TNF alpha, IFN gamma (Th 1). High rate of epithelial apoptosis is seen (Zeissig et al., 2007). Observation of thickness in mucus layer accompanied with abnormal PTM's of mucins (Strugala et al., 2008). Lack of expression of Human beta defensin 2&3 (Wehkamp et al., 2002). Expression of antiprotease are also reduced (Schmid et al., 2007).

#### Ulcerative Colitis (UC)

High levels of Claudins-2 expression (Luettig et al., 2015) and redistribution of Occludins, Claudins like−1,-4,-7. Cytokines mainly includes IL-13 and TNF alpha (Th 2) (Heller et al., 2005). This has also shown high apoptotic foci in epithelium. Mucus layer is almost diminished, very low goblet cells (Strugala et al., 2008), lowered expression of MUC2 &3 (Wibowo et al., 2019). Presence of HBD-2,3&4 (Fahlgren et al., 2004), increased levels of antiproteases (Schmid et al., 2007).

#### Cardiovascular Disease (Atherosclerosis)

Clinal studies on the patients suffering from atherosclerosis had altered junctions indicating a relation between cell junctions and atherosclerosis. Tight junction pathway was upregulated which significantly upregulates the expression levels of claudin−1, occluding, and ZO-1. Connexin 43 (Cx43) and 46 were also significantly increased. Earlier published study enlightens the fact that upregulated Cx 43 lead to monocyte adherence to vascular endothelial cells progressing further into atherosclerosis (Yuan et al., 2015). Cx 45, 46 exhibit synergetic associations and follows the similar pattern as Cx 43. Altered tight junction protein expression led to changed phosphorylation states. Regulation of the expression of ZO1 and occludin via myosin kinase inhibitor and its phosphorylation improved the atherosclerosis state in high fat fed rabbit model (Zhu et al., 2013). Proteins involved in SMAD pathway and TGF-β/endoglin levels were lowered in patients with atherosclerosis (Chen et al., 2017).

#### Gastric Cancers

Tight junctions have played specific role in cancer development (Table 1).

**Table 1:** Summarizes the role of various TJ in cancer.

**Type of cancer**	**Downregulation**	**References**	**Upregulation**	**References**
Colorectal carcinoma	Claudin 4,7 ZO- 1 Claudin 8	Suren et al., 2014 Kaihara et al., 2003 Grone et al., 2007	Claudin 2 Claudin 1 Occludins Claudin 12	Buhrmann et al., 2015 Sun et al., 2015 Grone et al., 2007
Gastric cancer	Claudin 17	Gao et al., 2013	Claudin 1 Claudin 4	Huang et al., 2014 Hwang et al., 2014
Hepatocellular carcinoma	Claudin 11 Claudin 14	Németh et al., 2008 Akizuki et al., 2017	Claudin 1 ZO-1 Claudin 11	Suh et al., 2013 Ram et al., 2018
Breast cancer	Claudin 1 ZO 1 ZO 2	Kim et al., 2008 Morohashi et al., 2007 Hoover et al., 1998 Chlenski et al., 2000	Claudin 3 Claudin 4 Claudins 20	Jaaskelainen et al., 2018 Jiwa et al., 2014 Martin et al., 2013
Lung cancer	Claudin 18 Claudin 7	Akizuki et al., 2017 Kudinov et al., 2016	Claudin 10	Zhang et al., 2013
Pancreatic cancer	ZO 2 ZO 1	Chlenski et al., 1999 Hoover et al., 1998	Claudin 4	Long et al., 2001
Renal cancer			Claudin 16	Buhrmann et al., 2015

#### Diabetes Mellitus (Type 1)

Hypothesized mechanism supports that the high gluten diet favors the increased colonization of Bacteriodes (Fasano et al., 1991) causing dysbiosis and zonulin pathway activation (Mowat, 2003). Food antigens such as wheat gluten (gliadin) can bind to CXCR3 intestinal epithelia leading to MyD88-activated zonulin pathway which further can cause increase in intestinal permeability (Fasano, 2001). Zonulin pathway activation leads to higher level of zonulin release (Visser et al., 2009) which then bind to its receptor present at intestinal epithelium and disassembles the tight junctions and also causing the phosphorylation of occludin, ZO-1 and alterations in occludin-ZO-1 and ZO-1- proteins interaction and polymerization of actin proteins (Bjorkman et al., 1987a). Disbursement of tight junctions assembly allows the entrances of luminal antigens which are then managed by immune cells like antigen presenting cells of lamina propria and presented to T cells (Bjorkman et al., 1987b). These events later on leads to autoimmune diseases i.e., Type 1 diabetes (Cuvelier et al., 1990). These data demonstrate that loss of gut barrier integrity is not only a contributor in T1D pathogenesis but a factor in causing chronic DSS-colitis triggering T1D in TCR-tg BDC2.5XNOD mice that otherwise do not develop diabetes. BDC2.5XNOD mice do not become diabetic despite having circulating T cells specific for an islet antigen. Exogenous events like a viral infection causes the disturbance in islets environment and leads to inflammation leading to tissue damage (Horwitz et al., 1998). Disturbances like the bacterial translocation though the compromised gut barrier induces the islet damage.

#### Obesity

Direct association has been established between obesity and permeability of intestinal epithelium. Experiments on obese mouse model such as ob/ob mice and db/db mice have shown the signs of altered tight junctions (Brun et al., 2007). These homozygous mice possess an increased levels of proinflammatory cytokines INFγ, IL-6, IL-1β, and TNF-α in comparison to wild-type control mice. Studies had revealed that the diet induced obesity (DIO) also lead to reduced expression of TJ proteins (Cani et al., 2009; Suzuki and Hara, 2010). DIO also contribute in changing the gut bacterial population by effecting the tight junction permeability inducing LPS associated endotoxemia and increased inflammation (Cani et al., 2008; Rohr et al., 2019) which is one of the reason for resistance developed for insulin. Other studies have used leptin-deficient mice and have shown that gut microbiome dysbiosis also interrelated with reduced expression of ZO1, occludin and genes synthesizing mucin (Nagpal et al., 2018).

#### NAFLD

There is an intimate interaction between the gut microbiome and metabolism in liver. Gut derived substances are received by the liver through the portal system. Recent studies provide evidences that the bile acids produced by liver are taken up by bacteria (Ridlon et al., 2014). Studies have demonstrated that NAFLD patients have reduced barrier integrity (Miele et al., 2009). Compromised gut barrier leads to increased bacterial endotoxin (LPS) permeability, which then enters the portal system. LPS, bacterial outer membrane of a gram -ve bacteria which act as a effective inflammatory molecule when binds with corresponding TLR activates NFκB and MAPK signaling pathways (Yuan et al., 2017) induces inflammatory cytokines production such as IL-6 and TNF-α (Dai and Wang, 2015) which slowly progress to liver fibrosis and cirrhosis. It was proposed that a dysbiosed intestine might lead to endogenous ethanol production which can in turn interrupt the mucosal barrier and elevates the LPS levels in the systemic circulation (Malaguarnera et al., 2014).

### Management of Barrier Integrity During Pathological Conditions

**Figure 2:**
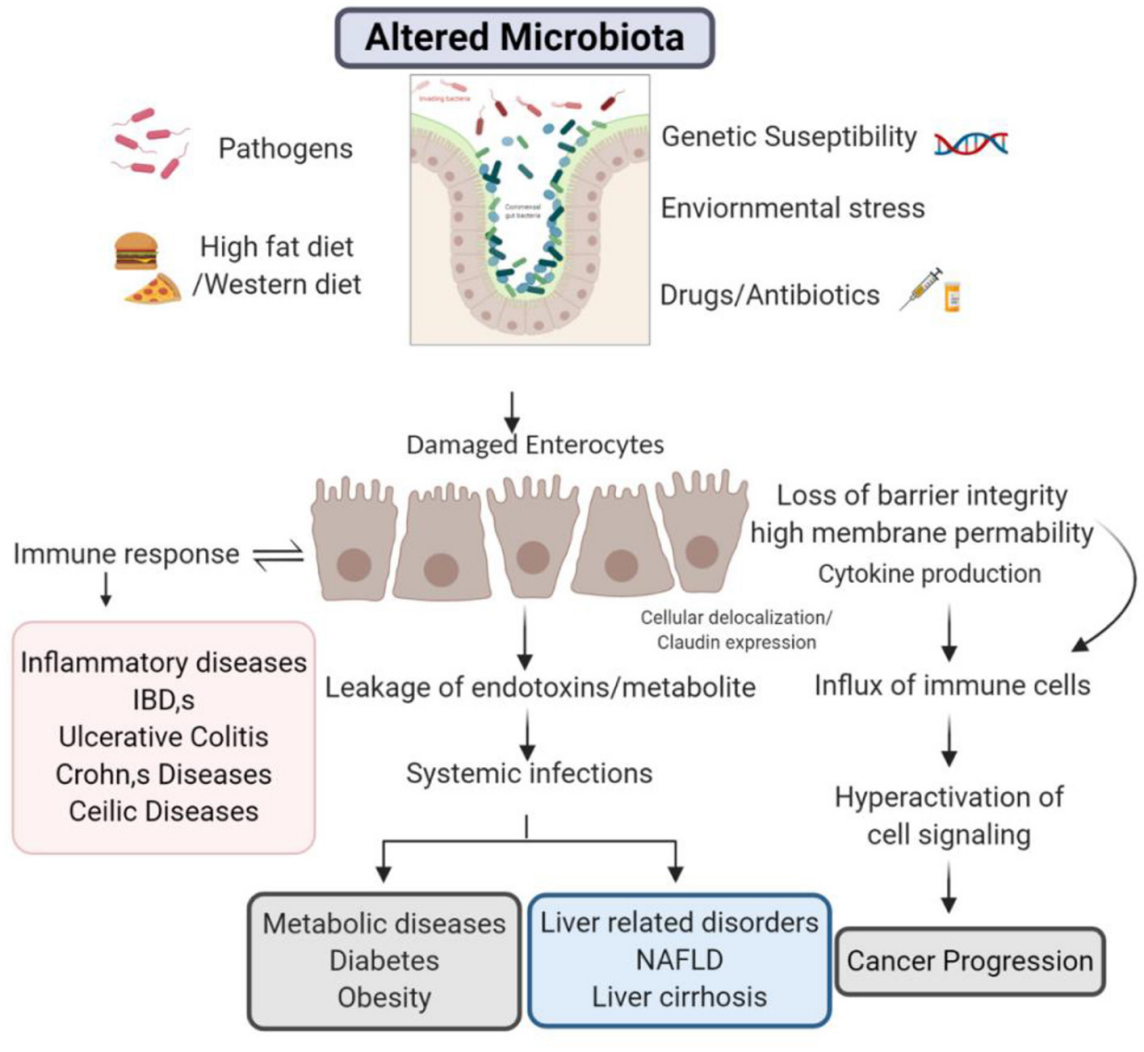
Barrier integrity during diseased conditions.

There is a recent attraction toward the role of gut barrier in disease pathogenesis. Concede intestinal barrier integrity is noticed in both systemic as well as intestinal diseases and other metabolic disorders discussed above. Although, the researchers are still trying to explore how the alterations in barrier integrity and can affect the pathogenesis of several diseases. Pathological or ecological elements may be the decisive role to seize healthy functionality and enhancement in the barrier permeability. In future, it is essential to appreciate the aspects that subsidize the barrier integrity damage under diseased circumstances (Figure 2).

Scientific evidences have shown the claudins play vital role in regulation of cancer. MAPK and PKC facilitated phosphorylation of claudin regulate the barrier functions (Bhat et al., 2019). PKA mediated phosphorylation allows the increased Mg^2+^ transport (González-Mariscal et al., 2008). Researches have demonstrated that claudins of colonic regions have perturbed phosphorylation in cases of colitis (Zhu et al., 2019). Blood vessel epicardial substance (BVES) functions in epithelial to mesenchymal transition through tight junction signaling pathways (Williams et al., 2011). Exposure to ozone in mice drastically increased claudin 3 along with cytokines and also reduced the levels of claudins 14 expression which establishes a co-relation between TJ and cancer (Kim et al., 2018). Claudin 2 overexpression was seen in patients suffering from colorectal cancer (Kinugasa et al., 2007) functions through EGFR signaling mechanism. Rabquer and colleagues have shown angiogenic role of JAM C (Rabquer et al., 2010). On the other hand, inactivation of p53 dependent pathway via notch 1 in HCT 116 could be reason for reduced apoptosis. Metastasis was seen in transgenic mice which exhibit increased expression of claudin 1 via notch signaling (Kim et al., 2007). PKC activation leads to claudin 1 overexpression resulting in invasion of cells via c-Abl-PKC signaling in liver cells (Yoon et al., 2010). Claudin 3&4 have been shown to enhance EMT in cancerous cells of ovary (Lin et al., 2013). ERK a kinase phosphorylates an array of proteins which might be involved in variety of functions like proliferation, survival, apoptosis, metabolism etc. Claudin 7 have been linked to contribute in metastasis of esophageal squamous carcinoma (Usami et al., 2006). EGFR and other several signaling pathways are linked with claudin 2 overexpression in lung cancer cell lines A549 (Ikari et al., 2012). Loss of barrier integrity is tightly associated with inflammation which in many cases progresses in cancer. Inflammation could be the reason for increased paracellular permeability and flux of immune cells which then contribute in release of proinflammatory cytokines (Al-Sadi et al., 2009). These proinflammatory cytokines in-turn induces the changes in TJ proteins in a loop and also promote survival of cancer cells. Influx of immune cells releases ROS which induces mutations in cells by causing DNA damage and might also inactivate mismatch repair (Kidane et al., 2014). Cancer is affiliated to immune cells by production of ROS, supporting angiogenesis and metastasis, all of this involves many pathways like COX-2, IL-6/STAT3, NF -kB, TNFα, Th17 signaling but there is still a need to revisit the links to get a better understanding about the TJs and their role in cancer.

## Role of Dietary Plant Components in TJs Maintenance

Present medication strategies in the health system include chemically synthesized antivirals, antibiotics, steroids, inhibitors and many more. From past two decades there are attraction toward natural compounds derived from plant parts from past 2 decades. Although, safety concern of such agents is parallelly emerging however, they are very cost effective and their availability in local region are the benefits of these new drugs. Phytochemicals is very wide term used to describe many compounds derived from herbal products or plants with therapeutic values toward health with low toxicity/side effects. Despite few clinical trials in patients, phytochemicals are generally recognized as safer substitute from the typical conventional remedies. Among all of them, most potential ones are terpenoids, saponins, organ-sulfides, alkaloids, lignans, and polyphenols. They possess abundant health benefits including detoxifying enzyme modulation, antimicrobial activities, immune system stimulation, antioxidant activities etc. thus possess lots of capability to intervene in therapeutic significance (Lee et al., 2018). In this review article, we aimed to represent the studies performed in this area, primarily concentrating on phytochemical exhibiting favorable effects in the intestinal barrier enhancement together with their mechanistic events. The following sections will be discussing the role of different phytochemicals, their possible mechanism of action, and prospects.

Certain biologically active compounds plays a pivotal role in upholding the tight junction integrity (Kovacs and Mela, 2006). Phytochemicals and their secondary metabolites are potentially used in reduce the paracellular transport through tight junction is extensively reported (Lee et al., 2018).

### Quercetin

Quercetin belong to flavonoid group and is reported to enhance the intestinal TJ barrier. The mechanism behind the action of Quercetin are transcription factors involved in enhanced expression of claudin 4 mediated by quercetin (Noda et al., 2014). Degraded products of quercetin such as 2,4,6,-trihydroxybenzoic acid and 3,4-dihydroxybenzoic acidalso enhances the expression of claudin 4 (Amasheh et al., 2009). Claudin 2 levels are reduced that restores the permeability induced by TNF-α by addition of quercetin *in-vivo* and *in-vitro* (Amasheh et al., 2012). Reports published focus on the reduction of inflammation and restoration of occludin and claudin (Shigeshiro et al., 2013). PI-3 Kinase cell signaling is inhibited by quercetin in the concentration dependent manner, since it is already established that this signaling is involved in cell proliferation (Agullo et al., 1997). ZO2, occluding and claudin 1 assembles in stimulus of quercetin by inhibiting the PKCδ activity in Caco-2 cells (Suzuki and Hara, 2009). 200 and 100 μM of quercetin have shown enhanced appearance of claudin-1&−4, ZO-2, occludin through the reduction of PKG (Amasheh et al., 2008) and PKCδ (Elias et al., 2009) activity, respectively on caco-2 cell lines. These studies directly correlate the effects of quercetin to maintain the barrier integrity. Quercetin significantly suppress MLCK activity (Suzuki and Hara, 2009). As evident by the above studies, quercetin regulates PKC activity and enhance the expression levels of TJs proteins indicating its tremendous potential in management of membrane integrity associated disorders.

### Berberine

Berberin is an isoquinoline alkaloid used an ancient Asian medicine to treat gut allied problems (Zhang et al., 2011). Evidently the effects of berberine on membrane integrity has been studied cell models (*in-vitro)* and animal models (*in-vivo*). At the time of microbial sepsis membrane integrity is lost affecting specific TJ proteins, such as the integral membrane protein occludin (Furuse et al., 1993), zonula occludens (ZO) (Stevenson et al., 1986), and claudin family members (Furuse et al., 1999) and the opening of tight junction is controlled by myosin light chain kinase phosphorylation (Turner, 2006). Experiments on rat models demonstrated that LPS induced sepsis is lowered when MLCK is inhibited. Involvement of NFκB allows the increased expression of MLCK that stimulate the breakdown of TJ's (Ye et al., 2006). Other *in-vitro* studies have confirmed that berberine functions to the lower the expression of inflammatory genes by reducing NFκB signaling. Berberine mediate the inhibition by working on IκB kinase and stabilizing IκB factor. Type 2 diabetic rats have shown improvements when supplemented with berberine, significant improvements were seen on intestinal permeability (Gong et al., 2017). Lee et al. has shown that TJ health was enhanced when Caco-2 cell lines were supplemented with 100 μM of berberine (Lee et al., 2018). There are many studies conducted on berberine in different model systems showing their strong anti-inflammatory property. Berbarine supplementation increase the expression of major proteins of TJ complex, thus regulating the membrane permeability.

### Curcumin

Curcumin is a polyphenolic compound which is found abundantly in turmeric commonly used as spices in Asian countries. It has anti-tumoural, healing and anti-inflammatory and anti-oxidative effects. It has been reported to show positive effects on reproductive, respiratory, cardiovascular and digestive health (Ali and Soheil, 2013). In a study when the caco2 monolayers were stimulated with proinflammatory cytokines like IL-6 and TNF apha (Ma et al., 2005), through NFκB signaling deteriorated the function of TJ but the pretreatment of curcumin abrogated this effect (Joe et al., 2004; Wang et al., 2017). Another mechanism of curcumin involves the suppression of IL-1β p38 signaling leading to inhibition of p38/(Al-Sadi and Ma, 2007) MAP Kinase resulting in MLCK inactivation, so phosphorylation did not take place on MLC maintaining the structural integrity of intestinal TJ (Gong et al., 2017). It is found that curcumin has capacity to reduced colonic histopathological score and colonic myeoperoxidases activity. It is also observed that phenolic compounds, and curcumin or rutin, can restored the length of colon significantly, in DSS treated models. These compounds can prevent mucosal injury and reduced neutrophil infiltration, perhaps due to antioxidative activity and inhibition of NF-κB activation (Wang et al., 2017). Curcumin is very popular from old days and till now it has been used in various medicinal formulation. The above-mentioned studies are strong enough to claim its role in clinical gastrointestinal complication. There is urgent need to explore the functionality of such compounds in systematic way to meet the requirements of multi targeted herbal drugs.

### Kaempferol

Kaempferol belongs to flavonol and isabundant in common fruits and vegetables. They possess voluminous amounts of anti-oxidant and anti-inflammatory (Calderón-Montaño et al., 2011) components. Kaempferol is known to have positive effect in building the barrier integrity and TEER (Suzuki et al., 2010). A study demonstrated that kaempferol when given at concentration of 100 μM increased the TJ proteins like ZO1&2, Occludin, and Claudin 1, 3&4. It affects the organization of TJ proteins (Suzuki and Hara, 2010). Some studies demonstrate that it is also responsible for redistribution of lipid microdomain proteins like flotillin1 and caveolin1 (Nusrat et al., 2000; Suzuki and Hara, 2010) which are associated with TJ membrane integrity. Researchers demonstrated that Kaempferol swiftly and significantly elevated the TEER values of the Caco-2 cells, but surprisingly they did not disturbed the membrane permeability. However, due to increase in expression of TJ proteins there is advancement in the cell. In the same study, researchers observed that Kaempferol improved the expression levels of claudin-4, which is reported to decreases Na^+^ permeability (Suzuki et al., 2010). Clinicians have observed that psychological/physiological stresses and irritable bowel disease can lead to secretary diarrhea due to irregularity in intestinal ion secretion, including paracellular Na^+^ secretion (Suzuki et al., 2010; Calderón-Montaño et al., 2011). Kaempferol administration might provide an mitigative effect on diarrhea in such situations. Such findings shows that kaempferol might has capability to regulate the dysfunctions related to membrane permeability.

### Genistein

Genistein is an isoflavone compounds and is a known inhibitor of protein tyrosine kinases (Akiyama et al., 1987). Phosphorylation of particular proteins response to arrangement and functioning of the tight junction (Sakakibara et al., 1997). Some studies demonstrate that the pretreatment of Caco-2 (Rao et al., 1997) enterocytes for time duration 30 min with 300 μmol/L genistein enhanced a H_2_O_2_-induced lowered TEER; this dose dependent effect. 300 μmol/L of Genistein blocked the opening of TJ barrier which prevented the influx of enteric bacteria (Lee et al., 2018). 300 μM of genistein reduced c-Src kinase induced oxidative stress and inhibited the phosphorylation of tyrosine present within the TJ proteins including occluding, zona occluden, E-cadherin and beta catenin which stabilized TEER. Combined treatment with a xanthine oxidase and xanthine induces the oxidative stress and elevate the labeled mannitol uptake in the caco-2 cell monolayers which represents the dysfunction junctional barriers could be reverted by the administration of genistein (Rao et al., 2002). A separate study shows that genistein showed a protective role against DSS fed mice and improved the barrier function. It also lowered cytokine expression while reducing the colonic inflammatory responses (Zhang et al., 2017). Genistein has also shown protection against acetaldehyde-induced model through intestinal TJ barrier function (Atkinson and Rao, 2001). It is assumed that the mechanism responsible for endotoxemia is due to the luminal acetaldehyde-induced damage of intestinal TJ barrier complex (Sheth et al., 2007). Genistein dose-dependent treatment prevented the acetaldehyde-induced intestinal permeability (Atkinson and Rao, 2001). Moreover, it is described that genistein improves the loss of intestinal TJ barrier function due to inflammation. However, the precise mechanisms underlying the genistein-mediated protective effects remain unclear and needs to be fully explore in future studies.

### Naringenin

Naringenin belongs to flavanone group and present in citrus fruits. They safeguard the TJ by elevating the occludins levels, JAM-A and claudins-3 in a DSS diet induced colitis mice (Azuma et al., 2013). Introducing Naringenin elevated TJ barrier, immunoblot analysis in the studies clearly depicted the association of TJ with cytoskeletal components. Claudin-4 expression levels were elevated with activation of claudin-4 promoter with Naringenin treatment (Noda et al., 2013). Assembly of TJ proteins is indeed a crucial factor against diseases like CD and UC (Azuma et al., 2013), researchers studies that in human caco-2 cells, feeding of naringenin protect the TJ proteins due to DSS mediated changes in colitis model (Noda et al., 2012). Naringenin is reported to increase the structural assembly in TJs when supplemented to caco-2 cells (Suzuki, 2020). Additionally, evidence shows intestinal bacteria can metabolized naringin in the proximal part of colon, which results in further smaller phenolics molecules (Stevens et al., 2019). Naringenin and their metabolites can influence the composition of intestinal microbial population and might employ beneficial effects on barrier functions and inflammation in intestine. Although the precise mechanisms of action are not well-defined more investigations in human subjects are warranted.

### Resveratrol

Resveratrol comes under stilbenoid group and is enormously present in grapes and peanuts. A study demonstrate that the effects induced by DSS fed colitis mice are alleviated by the diet rich in resveratrol and restoration of TJ proteins like ZO-2, occludin, JAM-A, claudins 3,4 and 7 was evident. Caco-2 treated with resveratrol have shown reduced production of IL-8 resulting in lowered infiltration of neutrophils and hence is at the center of resveratrol mediated (Mayangsari and Suzuki, 2018a). Resveratrol has shown alleviation for oxidative stress and IL-6 induced hyperpermeability (Azuma et al., 2013). Levels of IL-6 regulate the hyperpermeability of intestine epithelium as it increases claudin-2 expression that leading to pore formation via ERK1/2 and PI3K but introduction of resveratrol reduces the expression of IL-6 induced ERK 1/2 activation thereby lowering the expression of claudin 2 (Suzuki et al., 2011). Resveratrol showed a potential to regulate intestinal TJ barrier in dose dependant manner (50, 100, and 200 μmol/L) in *in-vitro* models (Mayangsari and Suzuki, 2018b). A study reports H_2_O_2_ facilitated loss of barrier integrity is due to the occludin expression upregulation via Tyr phosphorylation but the effects are restored by resveratrol (Mayangsari and Suzuki, 2018b). Others have reported to show the enhanced levels of c-Src regulating the phosphorylation of tyr residue 398 and 402 in occludin which in turn prevents the interaction of occludin with ZO-1 that results in destabilization of assembly of TJ (Elias et al., 2009). Even though information is still inadequate although it is assuring that resveratrol could be a useful agent for the treatment of gastrointestinal associated conditions.

## Future Perspective

New interrogation need to be arranged in future for clinical management of TJs associated diseases, since it is new area to emphasize safety concern of existing therapy as well as discovering alternative therapies, parallelly with the introduction of novel drugs. Both the clinical studies as well as basic research have provided the evidences for alignment between increase in intestinal permeability and several metabolic diseases like type 2 diabetes, celiac disease and irritable bowel syndrome. Till now the existing literature supports the usage of phytochemicals compounds in clinical trials and mentioned their beneficial effects with good safety profile. Dietary phytochemicals supplementation possibly might offer a potential remedy for these disease condition. Recently the researchers have developed the interest for studying the mechanisms involving phytochemicals and enhanced barrier integrity and reduction in gut permeability. However, in most instances, presented data were from studies in *in-vitro* models. Moreover, whether the phytochemical's effects are directly involved in the development and maintenance of epithelial barrier or it is mediated via enhancement of TJ proteins is unclear. Thus, detailed *in vivo* and more mechanism oriented studies are required, which may originate the development of plant based preventive or therapeutic approaches to combat the diseases linked with barrier dysfunction. In addition, the literature from available studies suggest that the polyphenols perform their beneficial activities in TJ regulation via cross talk between multiple pathways. Further studies are required in future to establish the mechanisms involved in the interactions between phytochemicals and the signaling pathways providing the defensive/protective actions on barrier function and formation of intestinal TJs. Studying these pathways and their interconnections will help to recognize the usage of these phytochemicals in the treatment and prevention of defects in intestinal barrier functions and the related diseases or complications. In this review, we explore the different direction in which TJs can influence human health and to pay more attention toward phytochemicals in terms of therapeutics agents. However, evidence-based information about the phytochemical usage is still lacking behind and more clinical studies should be elucidated to study their role and optimal administration are needed.

## Author Contributions

SS and PT: conceptualization, investigation, supervision, writing, reviewing, and editing. SP and SS: figure, formal analysis, and literature collection. All authors contributed to the article and approved the submitted version.

## Funding

PT was supported from programme grant funding from Department of Biotechnology, Govt. of India (Ramalingaswami Fellowship), Science and Engineering Research Board (SERB), Govt. of India, and CSIR-Indian Institute of Toxicology Research, Govt. of India. SS was supported by a post-doctoral fellowship women (PDFW) awarded by the University Grant Commission (UGC, India). Both the authors had a significant role in content, structure, and review of manuscript. The CSIR-IITR manuscript number is IITR/SEC/2021-2022/40.

## Conflict of Interest

The authors declare that the research was conducted in the absence of any commercial or financial relationships that could be construed as a potential conflict of interest.

## Publisher's Note

All claims expressed in this article are solely those of the authors and do not necessarily represent those of their affiliated organizations, or those of the publisher, the editors and the reviewers. Any product that may be evaluated in this article, or claim that may be made by its manufacturer, is not guaranteed or endorsed by the publisher.
